# Selective adsorption of 1,3-dimethyltrisulfane (DMTS) responsible for aged odour in Japanese sake using supported gold nanoparticles

**DOI:** 10.1038/s41598-018-34217-w

**Published:** 2018-10-30

**Authors:** Haruno Murayama, Yusuke Yamamoto, Misaki Tone, Takayuki Hasegawa, Moemi Kimura, Tamao Ishida, Atsuko Isogai, Tsutomu Fujii, Mitsutaka Okumura, Makoto Tokunaga

**Affiliations:** 1 Department of Chemistry, Kyushu University, Nishi-ku, Fukuoka 819-0395 Japan; 2 Research Centre for Gold Chemistry, Department of Applied Chemistry for Environment, Graduate School of Urban Environmental Sciences, Tokyo Metropolitan University, Hachioji, Tokyo 192-0397 Japan; 3 National Research Institute of Brewing, Higashihiroshima, Hiroshima 739-0046 Japan; 4 Department of Chemistry, Osaka University, Toyonaka, Osaka 560-0043 Japan

## Abstract

Gold (Au) nanoparticles (NPs) supported on SiO_2_ (Au/SiO_2_) were prepared by a practical impregnation method and applied as an adsorbent for 1,3-dimethyltrisulfane (DMTS), which is responsible for an unpleasant odour in drinks, especially Japanese sake. Compared with a conventional adsorbent, activated carbon, Au/SiO_2_ selectively reduced the DMTS concentration in Japanese sake without decreasing the concentrations of other aromatic components. DFT calculations revealed that the selective adsorption of DMTS occurred through the formation of a stable intermediate. The size of the supported Au NPs was controlled by the preparation conditions and determined from TEM observations and XRD measurements, and the size was ranged from 2.4 nm to 30 nm. Au/SiO_2_ having Au NPs with a diameter of 2.4 nm adsorbed DMTS the most efficiently. Smaller Au NPs showed better DMTS adsorption capabilities because larger amounts of Au atoms were exposed on their surfaces in the size range of this study. Langmuir-type monolayer adsorption and one-to-one binding of Au–S are proposed to occur based on an adsorption isotherm experiment. Even though significant differences of the fruity aroma score were not observed in the sensory evaluation between Au/SiO_2_ and activated carbon for this less aromatic Japanese sake, Au/SiO_2_ selectively decreased the DMTS concentration in the instrumental analysis.

## Introduction

**Figure 1:**
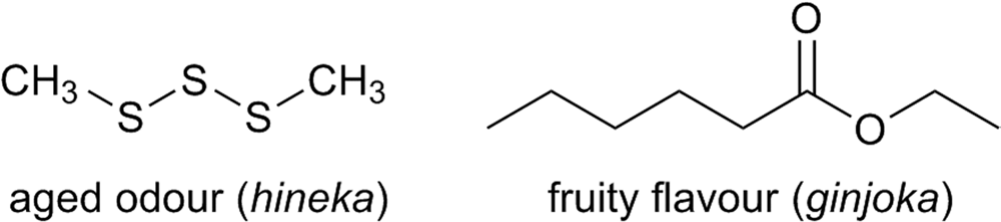
1,3-Dimethyltrisulfane (DMTS) and ethyl hexanoate (EH).

The control of flavour is one of the important issues for alcoholic beverages. Japanese sake contains many kinds of volatile compounds, including esters, alcohols, and acids. These compounds are mostly produced during alcohol fermentation and constitute the basic flavour of sake. A pleasant fruity aroma called “*ginjoka*”, which consists of esters such as ethyl hexanoate (EH) and 3-methylbutyl acetate, is also produced during fermentation. The flavour of Japanese sake also changes during storage. Although bio-catalytic reactions, including fermentation, are virtually stopped by heat sterilization, chemical reactions that generate several compounds not present in fresh sake take place^1^. A flavour perceived as a favourable mature aroma, called “*jukuseika*” in Japanese, emerges. On the other hand, an unfavourable aged odour, called “*hineka*”, can also emerge. Some of our authors revealed that sotolon is responsible for the mature aroma, while 1,3-dimethyltrisulfane (DMTS) is the major component of the unpleasant aged odour (Fig. 1)^2^. Sotolon creates a caramel- and maple syrup-like flavour, in contrast to DMTS, which has a sulphury, onion-like smell. A DMTS precursor molecule (1,2-dihydroxy-5-(methylsulfinyl)pentan-3-one, DMTS-P1) was identified in Japanese sake^3^. Recently, sake yeast with decreased DMTS-P1 productivity was developed^4^. DMTS is also involved in the off-flavour of beer^5^, whisky^6^, and wine^7^.

Many factors affect DMTS formation. For example, the fermentation temperature, which affects rice dissolution and yeast viability, has a large influence on DMTS formation during the storage of sake^8,9^. The sulphur content in the rice grain used to brew sake also affects the DMTS level after storage^10^. Suppression of DMTS formation by the addition of inorganic ions such as Mg^2+^ and SO_4_^2−^ to the fermentation mash of sake^11^ and beer^12^ has been reported. Copper, which is used in the distillation apparatus for whisky, the boiling kettles for beer, or the bottles for Japanese sake, causes the level of DMTS to both increase and decrease^13–16^. Controlling such brewing parameters will be effective in reducing DMTS formation; however, this control may not always be possible according to the priorities of the product design. For example, lower fermentation temperature, which suppresses the formation of DMTS after storage of sake, decreases the quantity of sake produced, because alcohol fermentation is decelerated at a lower temperature. The content of sulphur in rice grains varies according to not only the genetic background but also cultivation area. It is sometimes difficult to control DMTS only by brewing conditions; therefore selective adsorbents to remove DMTS in sake is required.

In the practical brewing process of Japanese sake, activated carbon has been traditionally used to reduce unpleasant flavours, perform decolouration, and adsorb impurities. However, activated carbon adsorbs a broad range of molecules into its micropores^2^. The use of activated carbon sometimes results in the production of an unscented sake. Although the DMTS concentration in Japanese sake can be decreased by less than 50% by activated carbon^2^, esters, including EH, which makes “*ginjoka*”, are also effectively adsorbed by activated carbon due to their hydrophobicity. Therefore, there is a demand for the selective removal of off-flavours without a decrease in the concentration of other components.

In this study, we first introduced noble metal nanoparticles (NPs) supported on metal oxides such as SiO_2_ to adsorb DMTS in a model solution. Because noble metals and sulphur compounds are known to have a high affinity, supported metal NPs have been applied in the adsorptive desulfurization of fuels to ppm levels^17,18^. Very few examples of noble metal adsorptive desulfurization of beverages exist^19^. The Ag supported on zeolite reduced the sulphur concentrations of dimethylsulfane, dimethyldisulfane, and DMTS in immature whisky. The performance of adsorbents was limited because a selective adsorption mechanism has not been clarified. Here we have investigated the mechanism of DMTS adsorption on the Au NPs with various diameters in model solution with various DMTS concentrations to develop the effective adsorbents. Since the organoleptic thresholds of sulphur compounds are extremely low, i.e., lower than ppb (≈ μg L^−1^) concentrations, we have carried out deep desulphurization of Japanese sake containing those concentration levels. Moreover, Au and SiO_2_, which are permitted for use in Japanese sake by laws, were applied to the selective adsorption of DMTS in stored Japanese sake without concomitant removal of esters.

## Results and Discussion

### DMTS adsorption from a model solution by supported noble metals

A model solution containing 4.7 mg L^−1^ of DMTS in ethanol was used to screen the adsorbents. Although this concentration is much higher than the threshold concentration of DMTS in Japanese sake (0.18 μg L^−1^), we chose this condition for the initial screening experiments to enable accurate analysis by normal FID-GC. Au, Pt, Pd, and Ru NPs supported on SiO_2_, whose secondary particle sizes were approximately 100 µm, with a metal/sulphur atomic ratio of approximately 6 (all 1 wt%) were examined for adsorption at room temperature without stirring and shaking (Table S-1). All metals except for Ru showed adsorption abilities. Au/SiO_2_ adsorbed almost all the DMTS within 24 h (Table S-1, entry 1). Au NPs, whose particle size was ranged from 3.0 nm to 10.9 nm, supported on several different kinds of oxide supports with secondary particle sizes of approximately 100 µm were tested (Table S-2, Fig. S-1). The results indicated that the smaller Au NPs showed faster and more extensive DMTS adsorption independent of the support material.

**Figure 2:**
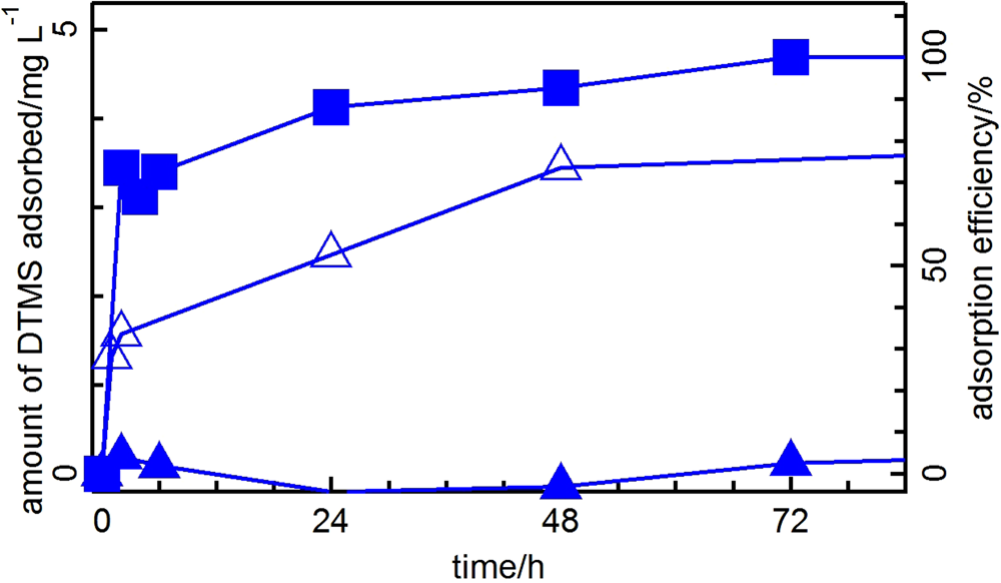
Time dependence of DMTS adsorption by Au/SiO_2_ having Au NPs with diameters of 2.4 nm (closed squares), 7.1 nm (open triangles), and 30.1 nm (closed triangles).

For further studies using the Japanese sake, we chose Au/SiO_2_ because the use of Au and SiO_2_ in Japanese sake brewing is permitted by law. SiO_2_ is commonly used as a clarification agent, and Au is used for decoration as gold foil. TiO_2_ could also support Au NPs as small as those supported on SiO_2_. Au/TiO_2_ showed a high DMTS adsorption performance, while for the removal of DMTS from Japanese sake, SiO_2_ is a more suitable support material than TiO_2_ owing to its acidity. The pH value of Japanese sake, which contains many carboxylic acids, is approximately 4.3^20^. Thus, SiO_2_, which is an acidic material, is expected to have no effect on the tastes of Japanese sake. On the other hand, TiO_2_, which possesses amphoteric and hydrophobic properties, adsorbs organic acids and esters, similar to activated carbon. However, the deposition of small Au NPs (<5 nm) on SiO_2_ as a support is difficult. Thus, we developed a novel practical preparation method for Au/SiO_2_, and a portion of the adsorption study was previously described^21^. The effect of the Au NPs particle size was explored. Au/SiO_2_ having Au NPs with a diameter of 2.4 nm adsorbed 88% of the total DMTS after 24 h and 100% at equilibrium (Fig. 2 and Table S-3, entry 1). As the size of the Au NPs increased, the amount adsorbed at equilibrium decreased. Au NPs with a diameter of 30.1 nm adsorbed only 1% after 1 day and quickly reached equilibrium. Moreover, gold foil (bulk) did not adsorb any DMTS under these conditions. The effect of temperature on the DMTS adsorption performances was investigated at 40 °C, 25 °C, and 10 °C (Fig. S-2). Au/SiO_2_ was suitable for use at room temperature, although the adsorption rate was slightly higher at higher temperature.

The average diameter of the Au NPs on SiO_2_ was almost the same before and after the adsorption experiments (Fig. S-3). The average diameters were 3.3 ± 0.9 nm before the experiment and 3.7 ± 1.0 nm after it. These results suggest that Au/SiO_2_ was stable in solution. In addition, the amount of Au ions leached into the solution after the adsorption experiment, which is listed in Table S-3, entry 1, was measured by microwave plasma atomic emission spectroscopy (MP-AES). The concentration of Au in the solution after filtration through a membrane filter (0.5 µm) was 4 mg L^−1^, namely, 3.2% of the total Au atoms leached into the solution. As mentioned below, the amounts of Au that leached in Japanese sake was below the detection limit of MP-AES because the concentration of DMTS in Japanese sake is lower than that in the model solution, and Au/SiO_2_ is stable even in the Japanese sake.

Competitive adsorption between DMTS and EH was carried out under similar conditions (Table 1). EH is a typical compound possessing a pleasant flavour. Using Au/SiO_2_, 100% of the total DMTS was adsorbed within 3 days, while 0% of the EH disappeared (entry 1). Au/Al-MCM-41 also adsorbed DMTS selectively with a slightly faster adsorption rate (entry 2). In contrast, Au/C adsorbed 21% of the EH as well as 100% of the DMTS, possibly due to the hydrophobic character of the pore interior. The Au NPs supported on SiO_2_-based materials selectively adsorbed DMTS from a solution mixture containing EH. The adsorption rate and amount were not reduced even with the coexistence of EH.

**Table 1:** Competitive adsorption between DMTS and EH by 1 wt% Au NPs supported on various supports^a^.

Entry	Support	Au particle size (nm)	% DMTS adsorbed after 24 h^b^	% DMTS adsorbed at equilibrium	Time to equilibrium (days)	% EH adsorbed^b^
1	SiO_2_	3.5	91	100	3	0
2	Al-MCM-41	2.5	99	100	2	0
3	C	6.6	82	100	4	21

^a^Adsorbents (52 mg, Au: 2.6 μmol) were added to an ethanol solution (4 mL) containing DMTS (4.7 mg L^−1^, Au/S atom ratio of 5.9), EH (5.2 mg L^−1^), and diglyme (3.1 mg L^−1^) as an internal standard and left at room temperature. ^b^The amount adsorbed was determined by GC analysis.

To understand the adsorption mechanism, an adsorption isotherm was created from several different initial concentrations of DMTS (Table S-4). This experiment was carried out with the Au NPs having a mean diameter of 3.5 nm supported on SiO_2_, where approximately 40% of the Au atoms were located on the surface. When the standard condition (DMTS = 4.7 mg L^−1^) was used (entry 1), the apparent Au/S atom ratio was 5.9, but the actual ratio to surface Au atoms was approximately 2.4; virtually 100% of the total DMTS was adsorbed and removed from the solution. When the initial amount of DMTS was doubled (entry 2), namely, the exposed Au/S ratio was approximately 1.2, 29% of the DMTS remained in solution at equilibrium. When the initial concentration of DMTS was increased to 28.1 mg L^−1^ (entry 4), 36% of the DMTS was adsorbed; under this condition, the Au/S and surface Au/S ratios were 0.96 and 0.4, respectively. In the solution with a DMTS concentration above 9.4 mg L^−1^, the Au NPs seemed to be fully covered by DMTS molecules.

**Figure 3:**
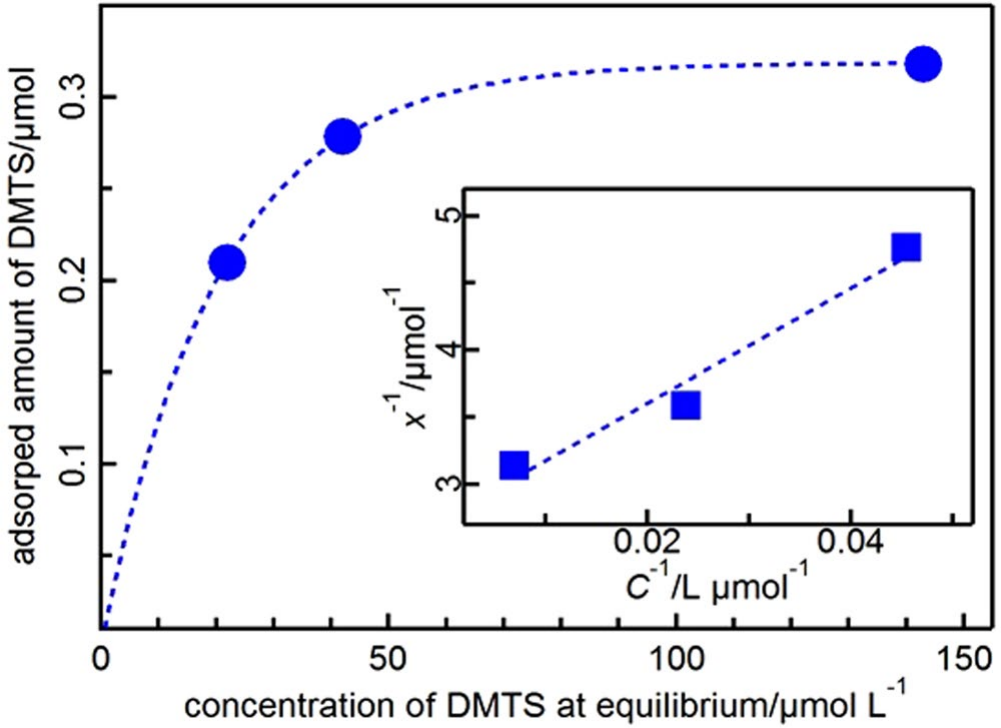
Adsorption isotherm and Langmuir plot (inset) of DMTS adsorption on Au/SiO_2_ (Au: 3.5 nm).

Figure 3 shows the adsorption isotherm plotted from the adsorbed amounts and concentration of DMTS at equilibrium listed in Table S-4, suggesting the occurrence of monolayer Langmuir-type adsorption. A Langmuir plot is given by Eq. 1.

1 $${x}^{-1}={(aK)}^{-1}{C}^{-1}+{a}^{-1}$$

In this equation, *x* is the amount adsorbed, *C* is the concentration of DMTS at equilibrium, *a* is the amount adsorbed at saturation, and *K* is the adsorption constant. The Langmuir plot derived from the adsorption isotherm (inset of Fig. 3) was well fitted by Eq. 1. The adsorption constant, *K*, and the amount adsorbed at saturation, *a*, were determined to be 64200 L mol^−1^ and 0.346 μmol, respectively, with 0.52 mg of Au under this condition.

The surface coverage, which corresponds to the ratio of DMTS adsorbed at saturation, can be calculated based on the Langmuir equation (Table 2). The coverage can also be calculated based on the number of atoms assuming that the proportion of Au atoms on the surface is 40% for an icosahedral Au NP and that the Au/S ratio for adsorption is 1. Interestingly, the coverage values are very close in each case, which means that the assumptions of monolayer Langmuir-type adsorption and one-to-one Au–S adduct formation are reasonable^22^.

**Table 2:** Coverage of the Au surface based on the Langmuir plot and the calculated number of Au surface atoms.

Entry	Initial conc. of DMTS (mg L^−1^)	Au/S atom ratio	% Coverage based on Langmuir plot	% Coverage based on number of Au surface atoms
1	9.4	2.9	64	62
2	14.1	1.9	81	81
3	28.1	0.96	92	93

### DFT calculation of DMTS adsorption on a Au_24_ cluster

**Figure 4:**
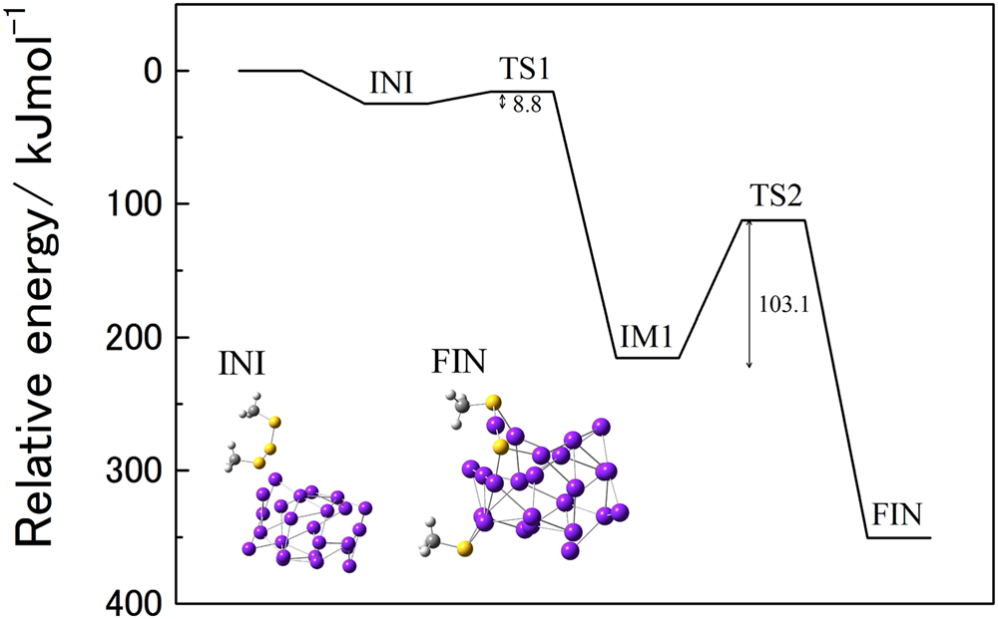
Energy diagram of the dissociative adsorption of DMTS on a Au_24_ cluster. The relative energy includes a zero-point energy correction.

DFT calculations were performed for DMTS adsorption on a Au_24_ cluster model system. The details of the calculation conditions are summarized in the Supplementary Information. The calculated reaction pathway is shown in Fig. 4. Based on this reaction pathway, the two S–S bonds in DMTS completely dissociated on the surface of the Au_24_ cluster, and the first and second activation barriers of this reaction are 10.2 and 103.3 kJ mol^−1^, respectively. Consequently, two CH_3_S groups and a S atom are generated on the Au_24_ cluster. A stapler-like Au–S–Au–S–Au bonding structure is formed on Au_24_ in the final structure (FIN) of the reaction pathway. This stapler structure is a well-known bonding mode for gold cluster complexes. Additionally, the adsorption energies of DMTS and EH on Au_24_ were compared. The structures and adsorption energies are summarized in Fig. S-4. The adsorption energies of these two substrates are almost the same, but the intermediate formed after the dissociation of the S–S bonds of DMTS on Au_24_ is much more stable than EH adsorbed on Au_24_. Thus, it could be concluded that the adsorptive decomposition of DMTS dominates the surface reaction of Au clusters.

### Instrumental analysis and sensory evaluation of Japanese sake

The utility of the supported Au NPs as adsorbents were examined with Japanese sake. Table 3 shows the results of instrumental analysis of DMTS and the primary flavour compounds in filtered Japanese sake. A control sake with aged odour contained 0.25 μg L^−1^ of DMTS, which is higher than the threshold value (0.18 μg L^−1^). The control sample (entry 1), also contained 1.4 mg L^−1^ of EH, which is one of the important components of a fruity aroma. This sake was treated with the supported Au NP adsorbents and activated carbon. All adsorbents decreased the concentration of DMTS; the Au NPs reduced the concentration from 0.25 to 0.01 and 0.03 μg L^−1^ (entries 2 and 3), while activated carbon reduced the concentration to 0.09 μg L^−1^ (entry 4). On the other hand, the activated carbon decreased the EH concentration from 1.4 to 0.6 mg L^−1^ (entry 4), whereas Au/Al-MCM-41 and Au/SiO_2_ adsorbed only small amounts of this compound. These results were consistent with the results obtained from adsorption experiments using the model solution shown in Table 1. Other aroma compounds, such as 3-methylbutyl acetate (another “*ginjoka*” component), ethyl acetate, and 3-methylbutan-1-ol, all of which are basic flavour components of sake, were hardly affected by the Au NPs. After the adsorption experiment listed in Table 3, entry 2, the concentration of Au in the Japanese sake was below the detection limit of MP-AES.

**Table 3:** Instrumental analysis of Japanese sake with aged odour and treated with adsorbents.

Entry	Sample	DMTS (μg L^−1^)	Ethyl acetate (mg L^−1^)	3-Methylbutyl acetate (mg L^−1^)	3-Methylbutan-1-ol (mg L^−1^)	EH (mg L^−1^)
1	control	0.25	34	0.9	123	1.4
2	Au/SiO_2_^a^	0.01	31	0.9	121	1.2
3	Au/Al-MCM-41^b^	0.03	33	0.9	121	1.3
4	activated carbon^c^	0.09	33	0.8	121	0.6

^a^5 g/500 mL adsorbent was added, left for 24 h, and then filtered. ^b^1 g/500 mL adsorbent was added, left for 24 h, and then filtered. ^c^0.5 g/500 mL adsorbent was added, left for 1 h, and then filtered.

The influence of supported Au NPs on sake flavour was also investigated by sensory evaluation (Table 4). Concerning the evaluation terms of odour, we examined four attributes; fruity aroma, aged odour, sulphur smell, caramel-like smell. DMTS is considered to contribute to sulphur smell and aged odour of Japanese sake. Fruity aroma is mainly attributed to EH and 3-methylbutyl acetate, and caramel-like smell in aged Japanese sake is mainly caused by sotolon. The adsorbents significantly decreased the aged odour and sulphur smell. For example, the sulphur smell score was 1.67 for the control (entry 1), 0.17 for both Au NP adsorbents (entries 2 and 3), and 0.33 for activated carbon (entry 4). The results well reflected the instrumental analysis (Table 3). However, significant differences were not observed in the fruity aroma in spite of the differences in EH levels between samples. This may be because the concentration of EH was too low. Actually, the sensory scores of fruity aroma were below 1 (1 = weak) for all samples. It is needed to use sake samples with higher level of EH to investigate the influence on fruity aroma. Similarly, the scores of caramel-like smell were low and not significantly different among samples. Sotolon is formed through a long period of storage (more than several years)^2^, and the test samples were not considered to contain enough level of sotolon to be perceived. We also examined taste attributes (body, dryness, smoothness, and aftertaste), and significant differences were not observed, which means that none of the adsorbents had a strong effect on the taste. It was demonstrated that Au/SiO_2_ selectively decreased the aged odour and the sulphur smell without significant changes in other sensory properties.

**Table 4:** Sensory evaluation of Japanese sake with aged odour and treated with adsorbents.

Entry	Sample	Fruity aroma (*ginjoka*)		Aged odour (*hineka*)		Sulphur smell		Caramel-like smell		Body		Amakara (dryness)		Smoothness		Aftertaste		Overall score	
		0: none		0: none		0: none		0: none		−2: light		−2: dry		−2: smooth		−2: dull		1: excellent	
		4: strong		4: strong		4: strong		4: strong		2: full		2: sweet		2: coarse		2: clean		5: faulty	
		ave.	stdev.	ave.	stdev.	ave.	stdev.	ave.	stdev.	ave.	stdev.	ave.	stdev	ave	stdev.	ave.	stdev.	ave.	stdev.
1	control	0.67^A^	0.82	2.17^A^	0.75	1.67^A^	1.21	0.50^A^	0.84	−0.33^A^	0.52	**−**1.00^A^	0.63	0.17^A^	0.98	**−**0.17^A^	0.41	3.83^A^	0.75
2	Au/Al-MCM-41^a^	0.50^A^	0.84	0.33^B^	0.52	0.17^B^	0.41	0.17^A^	0.41	−0.33^A^	0.82	−0.50^A^	0.55	0.17^A^	1.17	**−**0.33^A^	1.03	2.67^A^	1.21
3	Au/SiO_2_^b^	0.83^A^	0.75	0.83^B^	0.75	0.17^B^	0.41	0.00^A^	0.00	−0.33^A^	0.52	−0.50^A^	1.05	0.50^A^	0.55	−0.17^A^	0.41	2.67^A^	0.52
4	activated carbon^c^	0.67^A^	1.03	1.00^B^	0.63	0.33^B^	0.52	0.33^A^	0.82	−0.33^A^	0.82	−0.67^A^	1.03	0.67^A^	0.52	−0.67^A^	0.52	3.17^A^	0.41

^a^1 g/500 mL adsorbent was added, left for 24 h, and then filtered. ^b^5 g/500 mL adsorbent was added, left for 24 h, and then filtered. ^c^0.5 g/500 mL adsorbent was added, left for 1 h, and then filtered. ^A,B^Values with different letters are significantly different at *p* < 0.05 (*p* is the probability of obtaining the observed results, or more extreme, under the null hypothesis, i.e., no difference between samples) according to a Tukey-Kramer honestly significant difference (HSD) test.

## Conclusion

To remove one of the off-flavours caused by DMTS from stored Japanese sake without concomitant decreases in the concentrations of other aromatic compounds, we investigated the DMTS adsorption performances of noble metal NPs, especially Au NPs, supported on SiO_2_. Smaller Au NPs supported on metal oxide materials showed better DMTS adsorption performances in the size range of this study. Moreover, Au/SiO_2_ selectively adsorbed DMTS from a solution mixture containing EH. The mechanism of DMTS adsorption by the Au NPs was analysed using a Langmuir plot and DFT calculations. The Au NPs were covered by a monolayer of DMTS molecules, and one-to-one Au–S adducts were generated. The adsorption energies of DMTS and EH on a Au_24_ cluster were almost the same, while the generation of a more stable intermediate from the dissociation of the S–S bonds of DMTS led to the adsorption selectivity. Au/SiO_2_ also selectively reduced the DMTS concentration in stored Japanese sake. Significant differences in sensory evaluations were not observed in the fruity aroma for the less aromatic Japanese sake used in this study. The aged odour and the sulphur smell became weaker without significant changes in other sensory properties using Au/SiO_2_. It is expected that Au/SiO_2_ is more effective to use for drinks with highly aromatic concentration.

## Methods

### Preparation of adsorbent

Au/SiO_2_ was prepared by an incipient wetness impregnation method recently developed by us^21^. The typical preparation procedure is as follows: a Au–amino acid complex was obtained by mixing a HAuCl_4_•4H_2_O ethanol solution with a NaOH and amino acid ethanol solution. The mixture was left at −18 °C for 12 h, during which the Au–amino acid complex formed as a precipitate. The Au–amino acid complex was dissolved in 1.20 mL of water, and the solution was added to 0.99 g of SiO_2_ (CARiACT Q-15, Fuji Silysia Chemical Ltd.). After impregnation, the solid was calcined in air at 300 °C for 0.5 h. The details and a schematic of the preparation procedure are shown in Fig. S-5. The Au–β-alanine complex was used to form Au/SiO_2_ having Au NPs with mean diameters of 2.4 nm and 3.9 nm and Au NPs on the other supports. The Au–glycine complex^23^ was used to form Au/SiO_2_ having Au NPs with mean diameters of 3.5, 4.8, and 13.1 nm. The diameter of the Au NPs was changed by varying the preparation conditions, such as the volume of water and the calcination time. Larger Au NPs were obtained either when the volume of water was increased or when the calcination time was longer. The Au–β-alanine complex and the Au–glycine complex had essentially the same effect on the particle size. Au/SiO_2_ (Au: 30.1 nm) was prepared using HAuCl_4_•4H_2_O. Au/SiO_2_ (Au: 7.1 nm) and Au/C (Au: 6.3 nm) were purchased from Haruta Gold Inc. Activated carbon (Tokusen-shirasagi) was purchased from Takeda Kirin for the Japanese sake experiments. The mean diameters of the Au NPs on SiO_2_ listed in Table S-3 were determined from the FWHM values of the 111 peak obtained by XRD measurements based on the Scherrer equation. The average diameters of the Au NPs on SiO_2_ and Au/Al-MCM-41 were determined by transmission electron microscopy observation and found to be 3.9 ± 1.3 nm and 2.5 ± 1.0 nm, respectively (Fig. S-1). As described in the previous report^21^, the average diameter of the spherical particles observed in the TEM images was approximately the same as the crystallite size estimated from the XRD pattern, thus indicating that the Au NPs were supported on the SiO_2_ as small single Au metal crystals. The diameters determined by these methods are consistent in the case of the Au/SiO_2_.

### Adsorption experiments with a model solution containing 4.7 mg L^−1^ DMTS in ethanol

A 10 mL screw-top vial was filled with an ethanol solution (4 mL) containing DMTS (4.7 mg L^−1^) and diglyme (3.1 mg L^−1^) as an internal standard. Adsorbent was then added to the vial, which was left at room temperature. The concentrations of DMTS were determined by GC analyses. For the competitive adsorption experiments between DMTS and EH, the solution contained an initial EH concentration of 5.2 mg L^−1^.

### Adsorption experiments with a Japanese sake containing 0.25 μg L^−1^ DMTS

Typical procedure: A control sake sample was prepared by blending a 4/1 ratio of a sake stored at 40 °C for one month and a sake stored at 15 °C for 5 years. This blend was prepared because the sake stored at 40 °C for one month did not contain enough DMTS to be for this compound to be detected, whereas the sake stored for 5 years at 15 °C contained more DMTS. To this blend was added adsorbent, and the mixture was left at room temperature for 24 h. The adsorbent was removed by filtration using a 0.45 μm membrane filter. The filtrate was analysed by GC and sensory evaluation. Sensory evaluations were performed by 6 well-trained panellists.

Detailed information on the instruments, materials, Table S1 and S2, and Fig. S1 and S2 are available in the Supplementary Information.

## Electronic supplementary material

Supplementary Information
