# Supplementary material for: Selective adsorption of 1,3-dimethyltrisulfane (DMTS) responsible for aged odour in Japanese sake using supported gold nanoparticles
Source: Sci Rep. 2018 Oct 30;8:16064. doi: 10.1038/s41598-018-34217-w (PMC6207770; doi:10.1038/s41598-018-34217-w)
Supplement: Supplementary file 1 — Supplementary Information [file 41598_2018_34217_MOESM1_ESM.docx]

**Supplementary information**

**Selective adsorption of 1,3-dimethyltrisulfane (DMTS) responsible for aged odour in Japanese sake**

**using supported gold nanoparticles**

Haruno Murayama^1^, Yusuke Yamamoto^1^, Misaki Tone^1^, Takayuki Hasegawa^1^, Moemi Kimura^1^, Tamao Ishida^2^, Atsuko Isogai*^3^, Tsutomu Fujii^3^, Mitsutaka Okumura^4^ & Makoto Tokunaga*^1^

1. Department of Chemistry, Kyushu University, Nishi-ku, Fukuoka 819-0395, Japan

2. Research Centre for Gold Chemistry, Department of Applied Chemistry for Environment, Graduate School of Urban Environmental Sciences, Tokyo Metropolitan University, Hachioji, Tokyo 192-0397, Japan

3. National Research Institute of Brewing, Higashihiroshima, Hiroshima 739-0046, Japan

4. Department of Chemistry, Osaka University, Toyonaka, Osaka 560-0043, Japan

Correspondence and requests for materials should be addressed to A.I. (email: [isogai@nrib.go.jp](mailto:isogai@nrib.go.jp)) and M.T. (email: mtok@chem.kyushu-univ.jp)

**Table S-1.** DMTS adsorption from a model solution by 1 wt% *M*/SiO_2_^a^ (*M* = Au, Pt, Pd, and Ru)

| Entry | Adsorbent | Molar ratio of *M*/DMTS | % Adsorbed  after 24 h^b^ | % Adsorbed at equilibrium | Time to equilibrium (days) |
| --- | --- | --- | --- | --- | --- |
| 1 | Au/SiO_2_ | 17.7 | 91 | 100 | 3 |
| 2 | Pt/SiO_2_ | 17.4 | 66 | 100 | 7 |
| 3 | Pd/SiO_2_ | 19.2 | 22 | 27 | 7 |
| 4 | Ru/SiO_2_ | 20.1 | 0 | 0 | ― |

^a^ 1 wt% *M*/SiO_2_ were added to an ethanol solution (4 mL) containing DMTS (4.7 mg L^-1^, *M*/S atom ratio of 5.8–6.7) and diglyme (3.1 mg L^-1^) as an internal standard and left at room temperature. ^b^ The Amount adsorbed was determined by GC analysis.

**Table S-2.** DMTS adsorption from a model solution by 1 wt% Au/supports^a^

| Entry | Support | Au particle size (nm) | Molar ratio of Au/DMTS | % Adsorbed after 24 h^b^ | % Adsorbed at equilibrium | Time to equilibrium (days) |
| --- | --- | --- | --- | --- | --- | --- |
| 1 | SiO_2_ | 3.5 | 17.4 | 91 | 100 | 3 |
| 2 | Montmorillonite K10 | 6.1 | 17.2 | 58 | 84 | 7 |
| 3 | SiO_2_-Al_2_O_3_ | 10.9 | 17.2 | 43 | 74 | 7 |
| 4 | TiO_2_ | 3.0 | 17.2 | 100 | 100 | 1 |
| 5 | ZrO_2_ | 3.0 | 17.2 | 64 | 100 | 4 |
| 6 | Al_2_O_3_ | 7.5 | 17.2 | 43 | 78 | 7 |

^a^ Adsorbents were added to an ethanol solution (4 mL) containing DMTS (4.7 mg L^-1^, Au/S atom ratio of 5.7–5.8) and diglyme (3.1 mg L^-1^) as an internal standard and left at room temperature. ^b^ The amount adsorbed was determined by GC analysis.

**Table S-3.** DMTS adsorption from a model solution by 1 wt% Au/SiO_2_ with different Au particle sizes^a^

| Entry | Au particle size (nm) | % Adsorbed after 24 h^b^ | % Adsorbed at equilibrium |
| --- | --- | --- | --- |
| 1 | 2.4 | 88 | 100 |
| 2 | 3.5 | 91 | 100 |
| 3 | 4.8 | 65 | 100 |
| 4 | 7.1 | 53 | 76 |
| 5 | 13.1 | 39 | 54 |
| 6 | 30.1 | 1 | 1 |
| 7 | bulk | 0 | 0 |

^a^ 1 wt% Au/SiO_2_ (52 mg, Au: 2.6 μmol) were added to an ethanol solution (4 mL) containing DMTS (4.7 mg L^-1^, Au/S atom ratio of 5.9) and diglyme (3.1 mg L^-1^) as an internal standard and left at room temperature. ^b^ The amount adsorbed was determined by GC analysis.

**TEM images of the supported Au NPs**

**
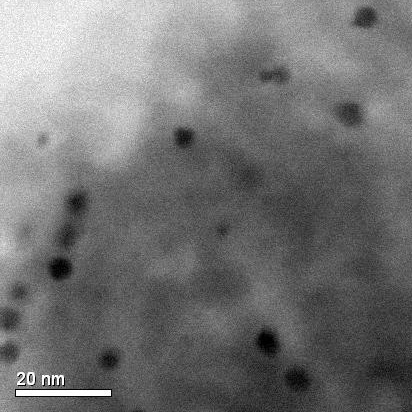
Au/SiO_2_ (3.9±1.3 nm)**

**Au/** **Al-MCM-41 (2.5±1.0 nm)**
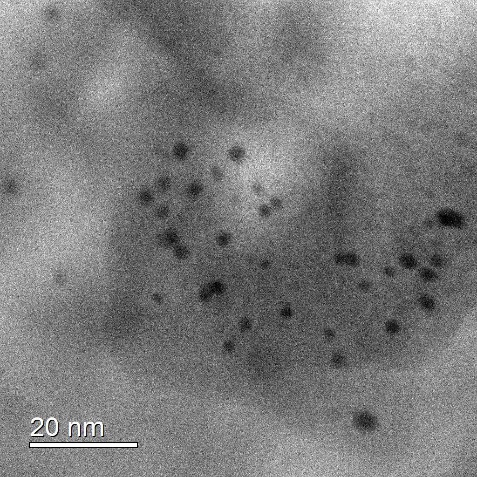

**Au/C (6.3±1.6 nm)**

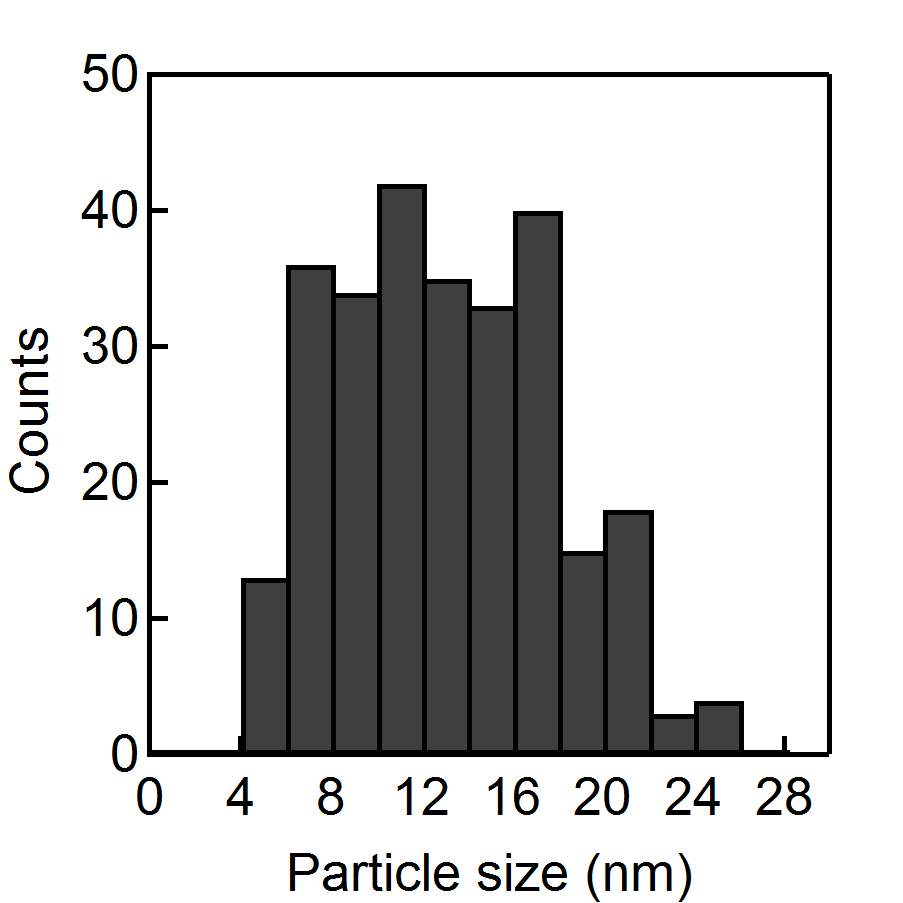

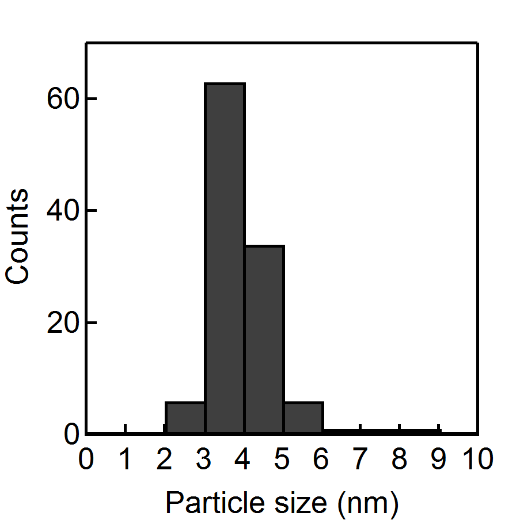

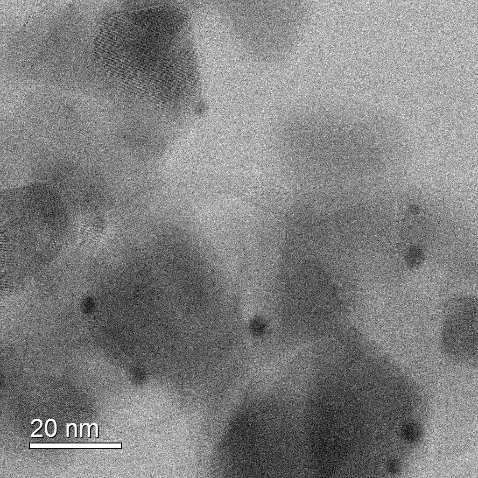

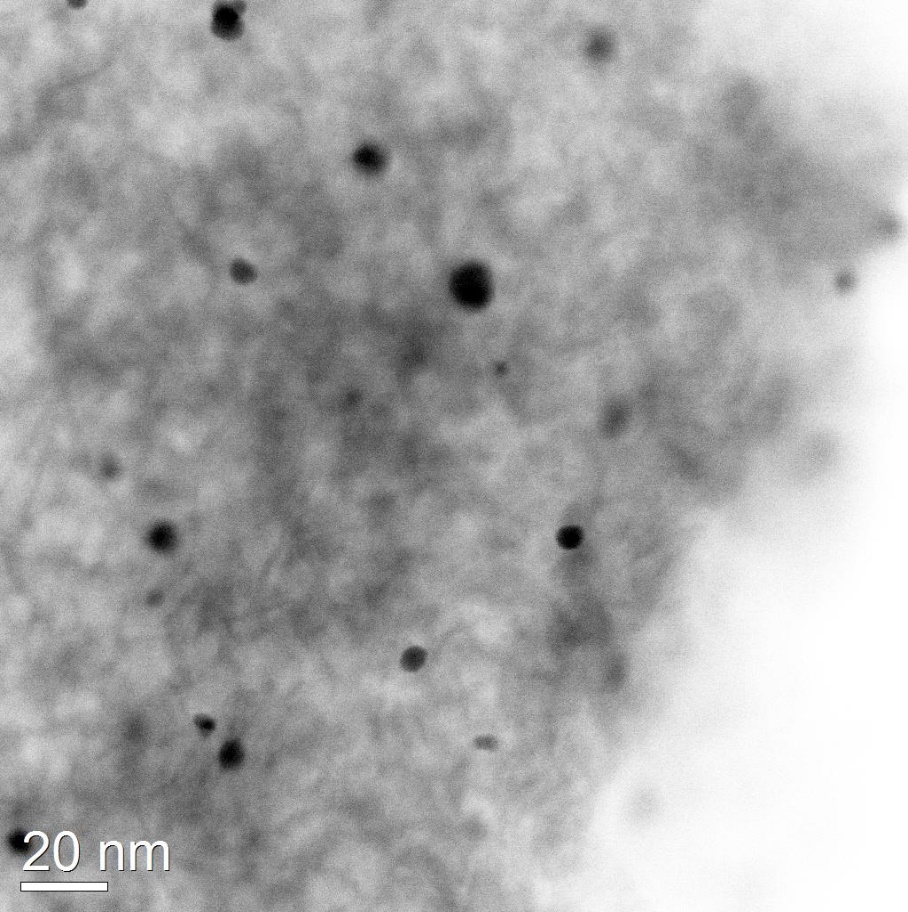
**Au/SiO_2_-Al_2_O_3_ (10.9±4.8 nm)**

**Au/TiO_2_ (3.0±0.9 nm)**

**Au/ZrO_2_ (3.0±0.9 nm)**
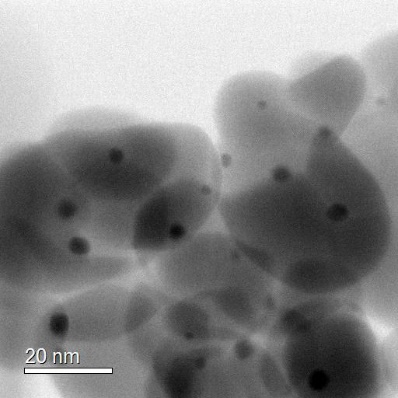

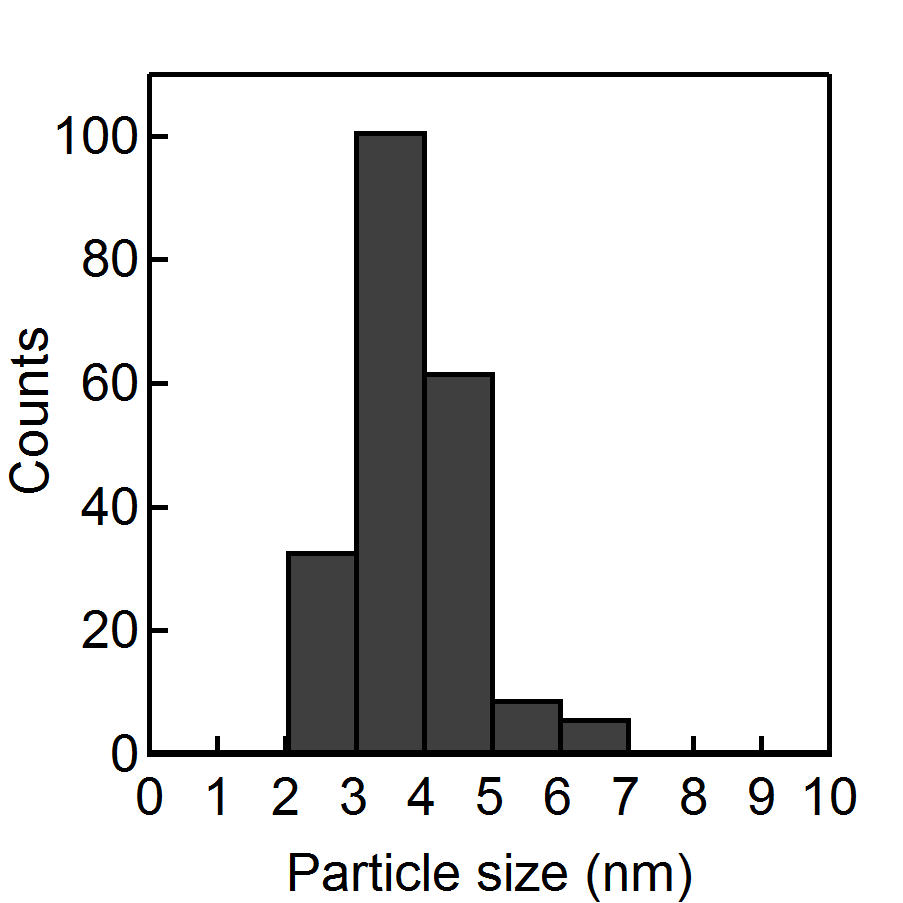


**Au/Al_2_O_3_ (7.5±2.7 nm)**


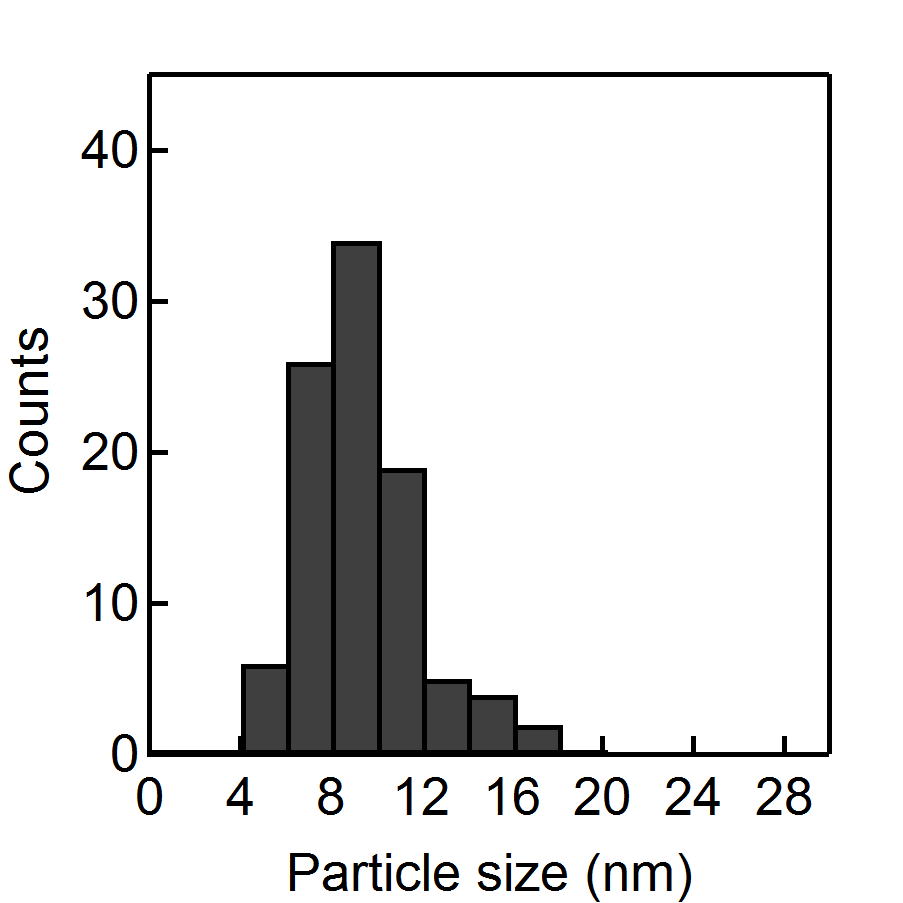


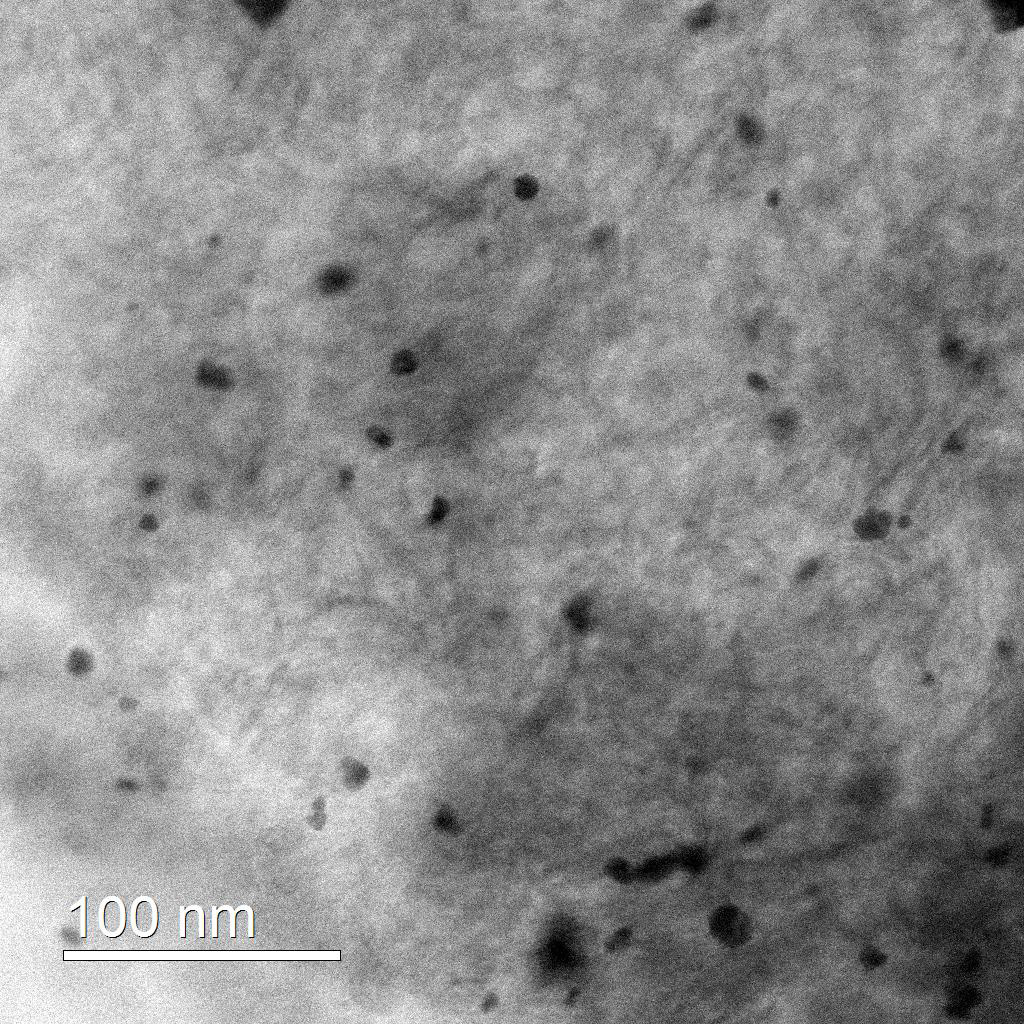


20 nm

**Figure S-1**. BF-STEM images of 1 wt% Au/SiO_2_-Al_2_O_3_, Au/TiO_2_, Au/ZrO_2_, and Au/Al_2_O_3_.

**Table S-4.** DMTS adsorption by Au/SiO_2_ with various initial concentrations of DMTS^a^

| Entry | Initial conc. of DMTS (μmol/L) | Au/S  atom ratio | % DMTS adsorbed at equilibrium^b^ | Adsorbed amount (μmol) | Conc. of DMTS  at equilibrium  (μmol L^-1^)^b^ |
| --- | --- | --- | --- | --- | --- |
| 1 | 37 (4.7)^c^ | 5.9 | 100 | 0.15 | 0 (0)^c^ |
| 2 | 74 (9.4)^c^ | 2.9 | 71 | 0.22 | 22 (2.7)^c^ |
| 3 | 112 (14.1)^c^ | 1.9 | 62 | 0.28 | 42 (5.4)^c^ |
| 4 | 223 (28.1)^c^ | 0.96 | 36 | 0.32 | 143 (18.0)^c^ |

^a^ 1 wt% Au/SiO_2_ (52 mg, Au: 2.6 μmol, Au mean diameter: 3.5 nm) were added to an ethanol solution (4 mL) containing DMTS (4.7–28.1 mg L^-1^, Au/S atom ratio of 5.9–0.96) and diglyme (3.1 mg L^-1^) as an internal standard and left at room temperature. ^b^ The amount adsorbed was determined by GC analysis. ^c^ In parenthesis is mg L^-1^.

**Temperature effects for the adsorption of DMTS**


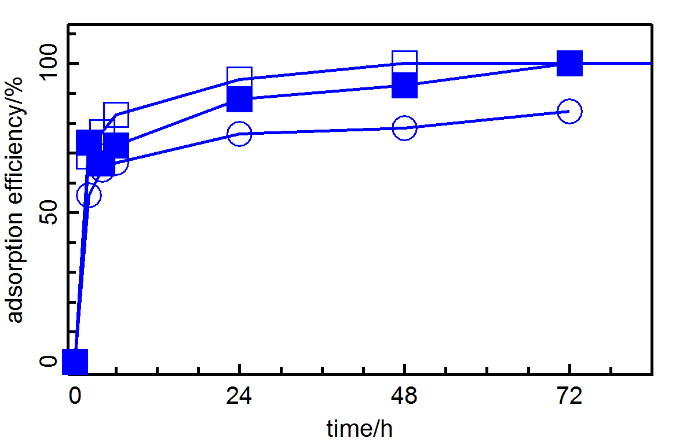


**Figure S-2. Time dependence of DMTS adsorption by Au/SiO_2_ having Au NPs with diameters of 2.4 nm at 40°C (open squares), 25°C (closed squares), and 10°C (open circles).**

**Stability of Au/SiO_2_**


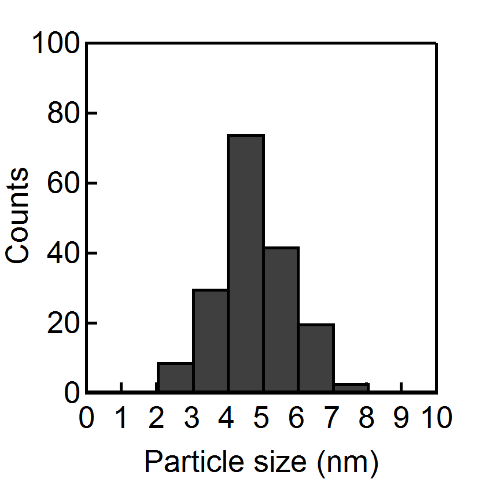

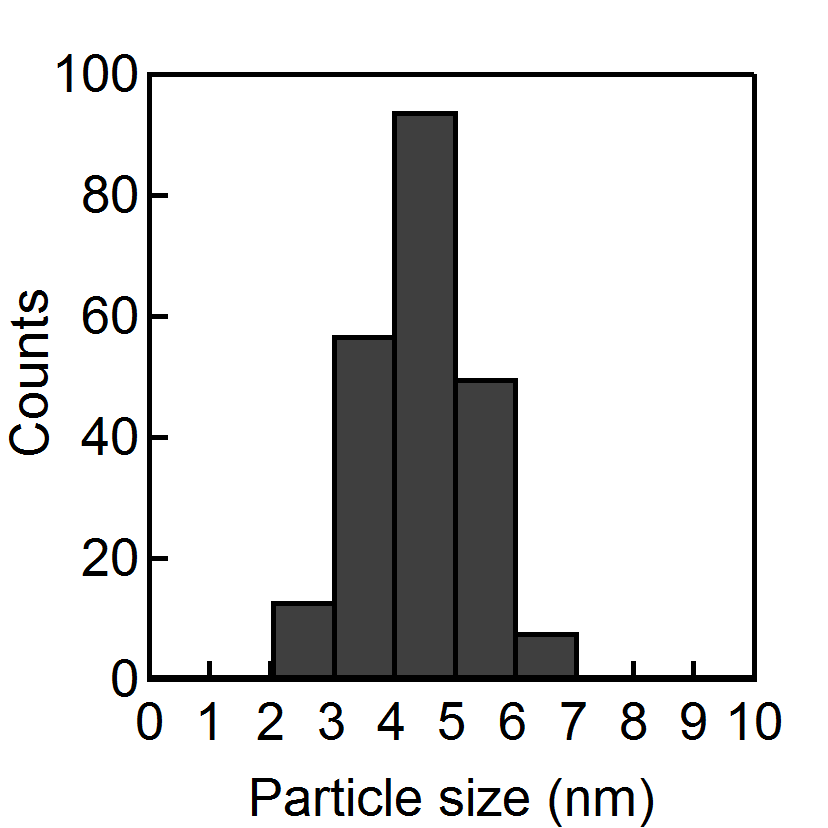

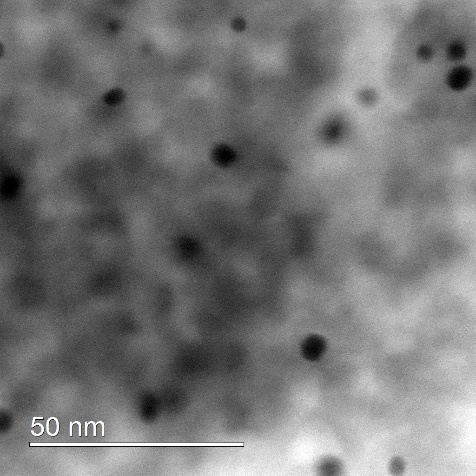


50 nm


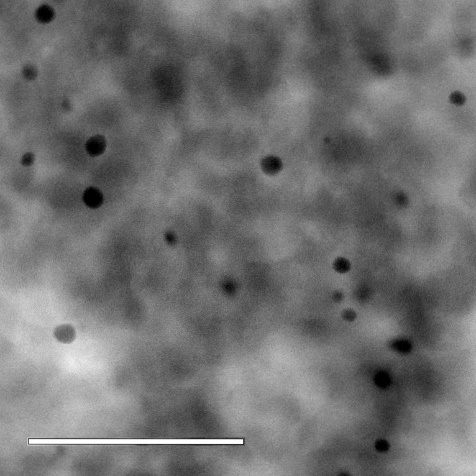


50 nm


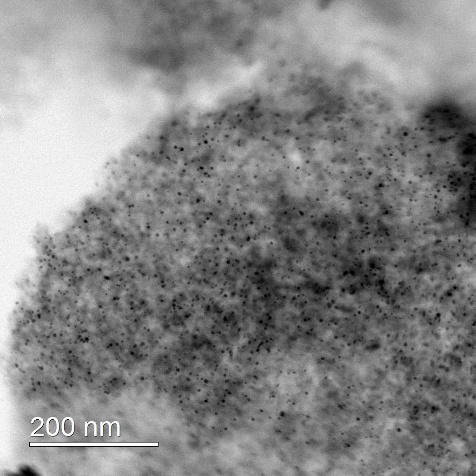

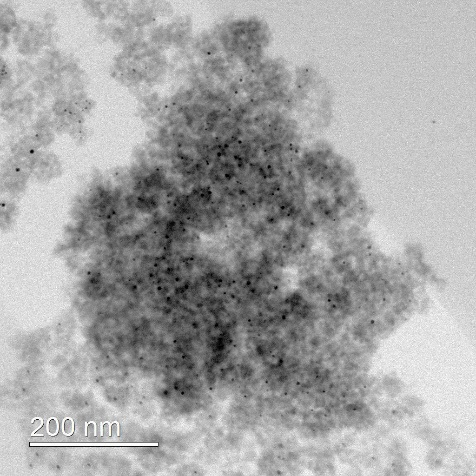


a

d

b

c

e

f

**Figure S-3.** BF-STEM images of Au/SiO_2_ before ((a) and (b)) and after ((d) and (e)) adsorption experiments. The size distributions of the Au NPs before and after the experiments are displayed in histograms (c) and (d), respectively. Their average diameters were 3.3 ± 0.9 nm and 3.7 ± 1.0 nm, respectively.

**Computational details**

To investigate the selective adsorption of DMTS by Au clusters, a Au_24_ cluster was used as a model system. DFT calculations were carried out on the structures of DMTS, EH, Au_24_-DMTS, and Au_24_-EH. In all DFT calculations, the PBE0 functional was used. The scalar relativistic effective core potential (ECP) with double-zeta basis sets (LANL2DZ) for all gold atoms and 6-31+G(d) basis sets were applied for the hydrogen, carbon, oxygen, and sulphur atoms. All the geometries of the model cluster systems were fully optimized. Vibrational analysis was performed on the optimized geometries of the transition states and local minima. To characterize the stationary points, frequency analysis was performed for all stationary points at this level. To establish the relevant species, the intrinsic reaction coordinate (IRC) pathway was also determined for all the transition states presented. The calculations were carried out using the Gaussian 09 software package.

**Calculation results**

(a) (b)


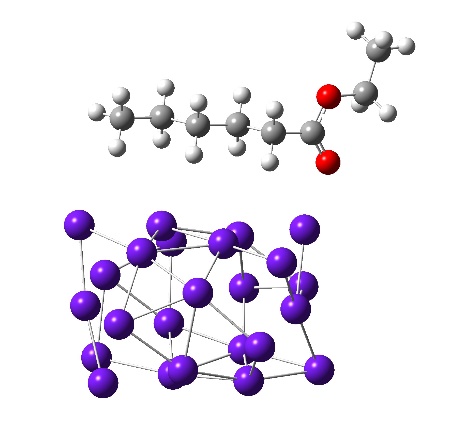

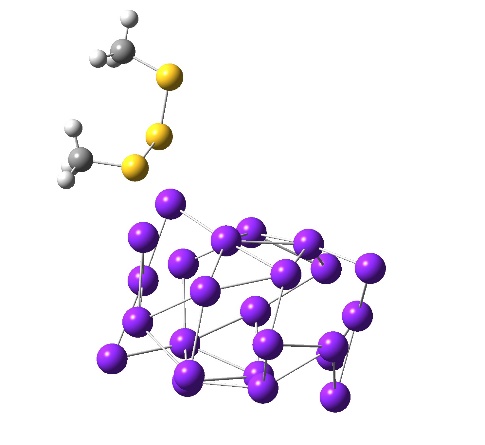


28.20 kJ/mol 28.10 kJ/mol

**Figure S-4.** Optimized structures of (a) DMTS and (b) EH adsorbed onto a Au_24_ cluster and adsorption energies with zero-point energy correction. The purple, yellow, grey, red, and white spheres represent gold, sulphur, carbon, oxygen, and hydrogen atoms, respectively.

**Details of the experimental methods**

GC analysis of a model solution containing 4.7 mg L^-1^ DMTS was carried out using an Agilent GC 6850 series II instrument equipped with an FID detector and a J&W HP-INNOWAX column (length: 30 m, inner diameter: 0.32 mm, film thickness: 0.25 µm).

The DMTS present in Japanese sake samples was analysed by stir bar sorptive extraction-GC/MS using an Agilent GC6890/MSD5973 instrument equipped with a thermal desorption system (Gerstel TDSA). Head-space GC analysis was carried out to measure the concentrations of ethyl acetate, 3-methylbutyl acetate, 3-methylbutan-1-ol, and EH in sake using an Agilent GC6890 instrument and 7694 headspace sampler.

XRD patterns were obtained with a Rigaku MiniFlex 600 diffractometer with Cu *Kα* radiation (*λ* = 0.15418 nm). The data were collected from 10 to 90° in 2*θ* using a step size of 0.02° and a scanning rate of 2°/min. The operating voltage and current were 40 kV and 15 mA, respectively. The phase of the components was identified by comparing the diffraction patterns with the ICCD PDF-2 powder diffraction file.

Scanning transmission electron microscopy (STEM) observations were performed at the Ultramicroscopy Research Center, Kyushu University, on a JEOL JEM-ARM200F system operating at 200 kV.

**Materials**

HAuCl_4_•4H_2_O, H_2_PtCl_6_•6H_2_O, Pd(NO_3_)_2_, and RuCl_3_•3H_2_O were purchased from Tanaka Kikinzoku, Wako Pure Chemical, Sigma-Aldrich, Inc., and Furuya Kinzoku, respectively. Au/SiO_2_ (Au: 7.1 nm) and Au/C (Au: 6.3 nm) were purchased from Haruta Gold Inc. Al-MCM-41 and montmorillonite K10 were purchased from Sigma-Aldrich, Inc. SiO_2_ (Q-15)_,_ SiO_2-_Al_2_O_3_ (JRC-SAH-1), Al_2_O_3_ (JRC-ALO-5), TiO_2_ (P-25), and ZrO_2_ (RC-100) were supplied by Fuji Silysia Chemical Ltd., Nippon Aerosil, and Daiichi Kigenso Kagaku Kogyo, respectively. Gold foil was purchased from Wako Pure Chemical.

Japanese sake samples (*honjozo-shu* and *tokubetsu junmai-shu*) were brewed in the National Research Institute of Brewing or purchased from a local market.

**Preparation of adsorbents**

The preparation of Au/oxide was described in the experimental procedure in the main text. Au/Mont (montmorillonite K10) and Au/SiO_2_-Al_2_O_3_ were prepared by an impregnation method using the Au–β-alanine complex. Au/TiO_2_, Au/Al_2_O_3,_ and Au/ZrO_2_ were prepared by deposition precipitation with urea (DP-urea).

**Preparation of 1 wt% Pt/SiO_2_**

Pt/SiO_2_ (Pt 1 wt%) was prepared by an impregnation method. SiO_2_ (0.99 g) was added to a H_2_PtCl_6_•6H_2_O (0.021 g, 0.05 mmol) aqueous solution, and the mixture was stirred for 30 min at room temperature. After impregnation, H_2_O was removed by evaporation and vacuum freeze-drying. The obtained catalyst was dried at 100 °C overnight and calcined in air at 400 °C for 4 h.

**Preparation of 1 wt% Pd/SiO_2_**

Pd/SiO_2_ (Pd 1 wt%) was prepared by an impregnation method. SiO_2_ (0.99 g) was added to a Pd(NO_3_)_2_ aqueous solution, and the mixture was stirred for 30 min at room temperature. After impregnation, H_2_O was removed by evaporation and vacuum freeze-drying. The obtained catalyst was dried at 100 °C overnight and calcined in air at 500 °C for 4 h. The PdO on SiO_2_ was reduced in a flow of H_2_ (10 mL min^–1^) at 300 °C for 4 h.

**Preparation of 1 wt% Ru/SiO_2_**

Ru/SiO_2_ (Ru 1 wt%) was prepared by an impregnation method. SiO_2_ (0.99 g) was added to a RuCl_3_•3H_2_O (0.026 g, 0.10 mmol) aqueous solution, and the mixture was stirred for 30 min at room temperature. After impregnation, H_2_O was removed by evaporation and vacuum freeze-drying. The obtained catalyst was dried at 100 °C overnight and calcined in air at 400 °C for 4 h. The RuO_2_ on SiO_2_ was reduced in a flow of H_2_ (10 mL min^–1^) at 300 °C for 4 h.

(b)

(a)

(c)

**Figure S-5.** Schematics of the experimental procedures; (a) synthesis of a Au–amino acid complex, (b) preparation of adsorbents, and (c) adsorption of DMTS.
